# Solid Tumor Opioid Receptor Expression and Oncologic Outcomes: Analysis of the Cancer Genome Atlas and Genotype Tissue Expression Project

**DOI:** 10.3389/fonc.2022.801411

**Published:** 2022-03-10

**Authors:** Amparo Belltall, Sheila Zúñiga-Trejos, Iris Garrido-Cano, Pilar Eroles, Maria Pilar Argente-Navarro, Donal J. Buggy, Oscar Díaz-Cambronero, Guido Mazzinari

**Affiliations:** ^1^ Perioperative Medicine Research Group, Instituto de Investigación Sanitaria la Fe, Valencia, Spain; ^2^ Departament de Anesthesiology, Hospital Universitari i Politécnic la Fe, Valencia, Spain; ^3^ Bioinformatics and Biostatistics Unit, INCLIVA Biomedical Research Institute, Valencia, Spain; ^4^ Breast Cancer Research Group, Molecular and Cellular Oncolgy Unit, Biomedical Research Institute, INCLIVA, Valencia, Spain; ^5^ Euro-Periscope: The Onco-Anaesthesiology Research Group (RG) of European Society of Anaesthesiology & Intensive Care (ESA-IC), Brussels, Belgium; ^6^ Department of Anaesthesiology and Perioperative Medicine, Mater University Hospital, University College Dublin, Dublin, Ireland

**Keywords:** opioid receptors, perioperative opioid, cancer, surgery, neoplasm, tumor, immunohistochemistry

## Abstract

**Background:**

Opioid receptors are expressed not only by neural cells in the central nervous system, but also by many solid tumor cancer cells. Whether perioperative opioids given for analgesia after tumor resection surgery might inadvertently activate tumor cells, promoting recurrence or metastasis, remains controversial. We analysed large public gene repositories of solid tumors to investigate differences in opioid receptor expression between normal and tumor tissues and their association with long–term oncologic outcomes.

**Methods:**

We investigated the normalized gene expression of µ, κ, δ opioid receptors (MOR, KOR, DOR), Opioid Growth Factor (OGFR), and Toll-Like 4 (TLR4) receptors in normal and tumor samples from twelve solid tumor types. We carried out mixed multivariable logistic and Cox regression analysis on whether there was an association between these receptors’ gene expression and the tissue where found, i.e., tumor or normal tissue. We also evaluated the association between tumor opioid receptor gene expression and patient disease–free interval (DFI) and overall survival (OS).

**Results:**

We retrieved 8,780 tissue samples, 5,852 from tumor and 2,928 from normal tissue, of which 2,252 were from the Genotype Tissue Expression Project (GTEx) and 672 from the Cancer Genome Atlas (TCGA) repository. The Odds Ratio (OR) [95%CI] for gene expression of the specific opioid receptors in the examined tumors varied: MOR: 0.74 [0.63–0.87], KOR: 1.27 [1.17–1.37], DOR: 1.66 [1.48–1.87], TLR4: 0.29 [0.26–0.32], OGFR: 2.39 [2.05–2.78]. After controlling all confounding variables, including age and cancer stage, there was no association between tumor opioid receptor expression and long–term oncologic outcomes.

**Conclusion:**

Opioid receptor gene expression varies between different solid tumor types. There was no association between tumor opioid receptor expression and recurrence. Understanding the significance of opioid receptor expression on tumor cells remains elusive.

## Introduction

Surgery remains a primary treatment for 70% of solid tumors (1) but analgesia after resection is challenging. Pain and nociceptive transmission involve neuronal networks with various receptor types that elicit either activation or suppression of the stimuli (2). Opioids are still the mainstay of postoperative pain management. Their primary site of action is the μ opioid receptor (MOR) which is expressed at various central nervous system (CNS) locations along the pain pathway. Opioid drugs activate MORs to suppress ascending nociception and enhance descending pain inhibition (3, 4). However, one-dimensional reliance on opioid medication has disadvantages. First, MOR is expressed in other tissues such as the brain stem and bowel, leading to undesired side effects such as respiratory depression, nausea, and ileus. Second, repeated, prolonged opioid administration results in hyperalgesia and has been linked to the ongoing problem of opioid dependence (5–8). Third, opioids suppress cell–mediated immunity and directly activate tumor angiogenesis, thereby potentially facilitating residual tumor cell spread (9).

Opioid drugs act as agonists not only at MOR but also at δ–opioid receptors (DOR), and both can be expressed by tumor cells (10). Cancer metastasis and proliferation may be associated with the activation of these opioid receptors through different pathways (11). However, MOR, DOR, and κ opioid receptors (KOR), in addition to opioid growth factor receptor (OGFR) and Toll–like receptor 4 (TLR4), have been shown to promote tumor cell migration (12–14). Previous studies aiming to elucidate the role of these receptors in cancer differ widely in their methodology, e.g., immunohistochemistry (IHC) or nucleic acid polymerase chain reaction (PCR) amplification, as well as in the studied samples, e.g., tissue or cell lines, and in targeted receptors (**eTable 1** in the **Supplementary Digital Content**) (15–24).

The evolution of genetic sequencing technologies and the drive to unravel the mechanisms underlying many human diseases has led to the appearance of large repositories of genetic data such as the Genotype Tissue Expression Project (GTEx) and the Cancer Genome Atlas (TCGA). The TCGA is a National Institute of Health (NIH) sponsored project that aims to discover significant cancer-causing genome alterations in large cohorts of tumors through large-scale genome sequencing (25, 26).

Our study objective was to analyse opioid receptor gene expression in tumors compared to normal tissue and to evaluate the association between this and long-term oncologic outcome, defined as overall survival (OS) and disease-free interval (DFI). Gene expression data was obtained from GTEx and TCGA.

## Materials and Methods

In this analysis we followed the recommendations on reporting results from observational studies (STROBE guidelines. https://www.equator-network.org/reporting-guidelines/strobe/).

### Population

We analyzed data from normal and tumor tissues from bladder, breast, colon, liver, salivary gland, esophagus, prostate, stomach, thyroid, lung, and kidney tumors.

### Data Collection

Unified GTEx and TCGA gene expression data for MOR, DOR, KOR, TLR4, and OGFR genes were obtained using an established technique (27) (Data record 3) for each tissue type. This dataset includes a strict selection of high-quality RNA-Seq samples processed with the same analysis pipeline and corrected for unwanted non-biological variation that affects comparative analyses. In addition, gene expression values were reported in Fragments per Kilobase Million (FPKM) units.

TCGA survival data were downloaded from the TCGA TARGET GTEx dataset deposited in Xenabrowser (https://xenabrowser.net/). We collected DFI and OS. The remaining clinical data for TCGA samples were obtained from TCGABiolinks (28). Information for GTEx individuals was directly downloaded from the GTEx project website (GTEx Analysis Release V8). Clinical information from the different sources and gene expression data in log2 (FPKM+1) scale were formatted and merged.

### Definitions

OS is the length of time from either the date of cancer diagnosis or the start of treatment and death from any cause. DFI is the length of time between primary cancer treatment and any signs or symptoms reappearance (29).

### Statistical Analysis

We used data of all available patients without formal sample size calculation. Also, as the purpose was to explore a pathophysiological hypothesis, we did not specify any *a priori* effect size. We reported continuous variables as median and 25^th^–75^th^ percentiles and categorical variables as numbers and percentages. Distribution was assessed by inspecting quantile–quantile plots, and log-transformation was carried out if the variable distribution violated the normality assumption. Finally, descriptive analyses were performed to summarize patient characteristics.

To assess the association between opioid receptor gene expression and type of tissue, i.e., control *versus* tumor, we fitted mixed logistic models introducing MOR, KOR, DOR, OGFR, and TLR4 genes as covariables, and primary tumor site as a random effect to consider the variability between different tumor sites. This model was fitted for tumors with data available for every receptor included in the analysis. We fitted a logistic model with all the receptor data available for each tumor type as a sensitivity analysis.

To assess the association between opioid receptor gene expression and DFI and OS, we fitted a mixed Cox model introducing MOR, KOR, DOR, OGFR, and TLR4 genes, age at diagnosis, and cancer stage as covariables. Primary site of tumor was a random effect to consider the variability between different tumor sites.

Statistical significance was set for two–tailed test at P<0.05. No missing values imputation and no correction for multiple comparisons was prespecified: thus, all the findings should be viewed as exploratory. All analyses were performed with R 4.0.3 (The R Foundation for Statistical Computing, www.r-project.org)

## Results

**Figure 1:**
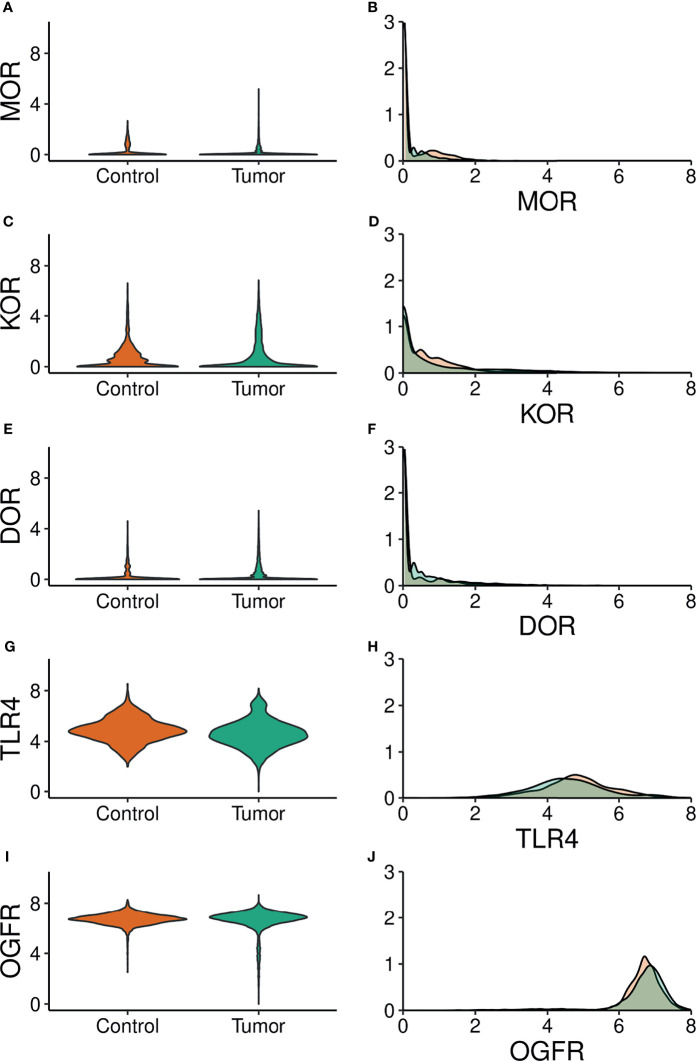
Violin (Left panels) and density (Right panels) plots of the expression of opioid receptor genes. Green: tumor samples. Orange: control samples. MOR, µ opioid receptor; KOR, κ opioid receptor; DOR, δ opioid receptor; TLR4, toll–like receptor; OGFR, opioid growth factor receptor. **(A, C, E, G, I)** Gene expression (Log scale) is on the y axis. **(B, D, F, H, J)** Gene expression (Log scale) s on the x axis.

**Figure 2:**
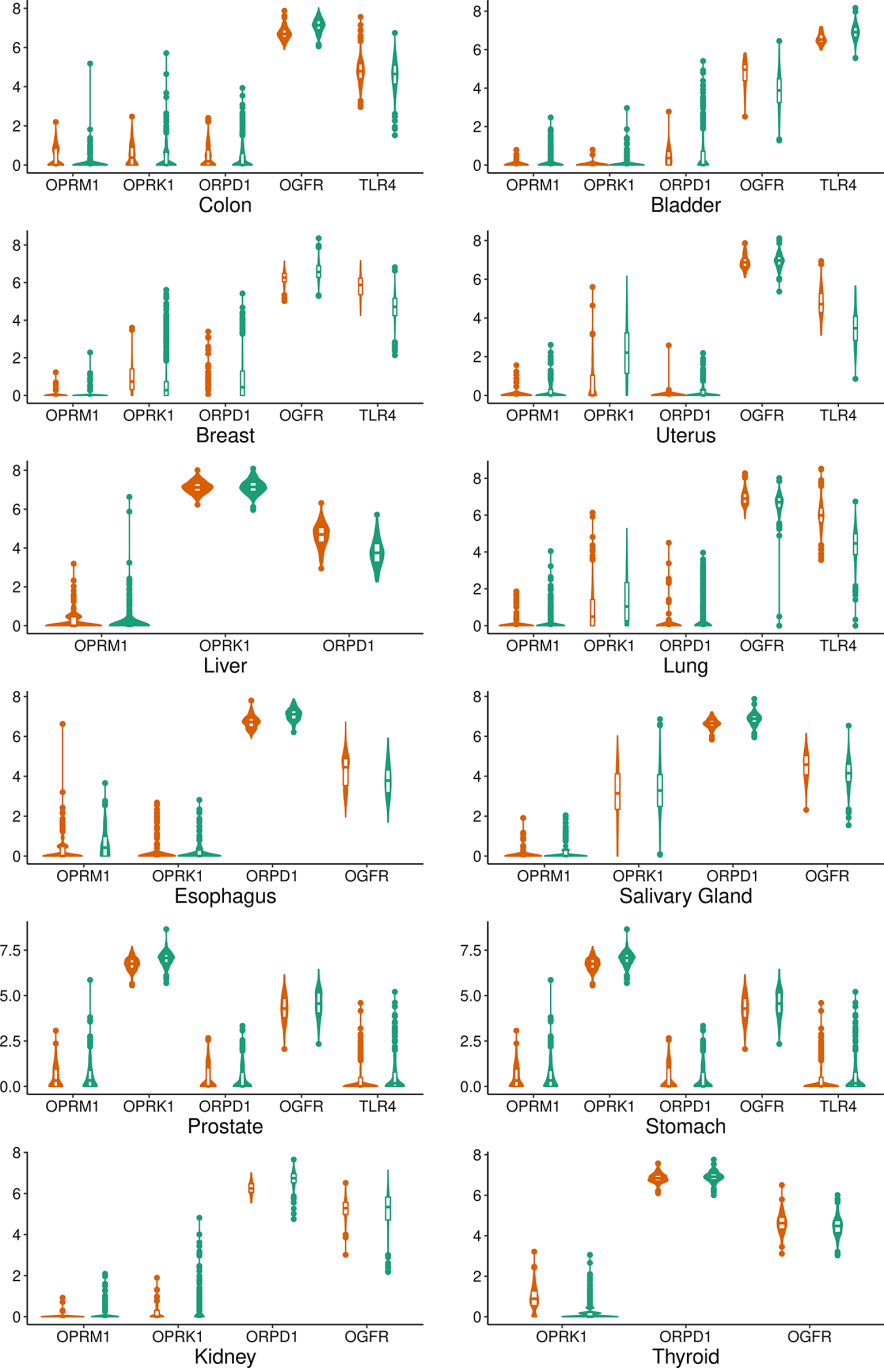
Violin and box plot graphs of the expression of opioid receptors genes by tumor type. Green: tumor samples. Orange: control samples. MOR, µ opioid receptor; KOR, κ opioid receptor; DOR, δ opioid receptor; TLR4, toll–like receptor; OGFR, opioid growth factor receptor.

We retrieved 8,780 tissue samples: 5,852 from tumor and 2,924 from normal control tissues, of which 2,252 and 672 were from the GTEx and TCGA repository, respectively. Sample characteristics are shown in **Table 1**. OGFR gene expression was highest while MOR gene expression was lowest, with comparable values between control and tumor samples on every overall gene expression. Median and percentiles values and violin and density plots (**Figure 1**) show considerable overlap. Opioid receptor expression by tumor primary site is reported in **Figure 2**.

**Table 1:** Clinical and tumor characteristics.

	Overall (N= 8780)	GTEx Normal (N= 2256)	TCGA Normal (N= 672)	TCGA Tumor (N= 5852)
**Tissue type** (tumor) % (N)	66.7 (5852/8780)			100 (5852/8780)
**MOR gene**	0 [0 – 0]	0 [0 – 0.3]	0 [0 – 0.3]	0 [0 – 0]
**KOR gene**	0.3 [0 –1.1]	0.4 [0 – 1.0]	0.4 [0 – 1.1]	0.2 [0 – 1.2]
**DOR gene**	0 [0 – 0.4]	0 [0 – 0]	0 [0 – 0]	0 [0 – 0.5]
**TLR4 gene**	6.7 [6.5 – 7.1]	6.8 [6.5 – 7.0]	6.7 [6.5 – 7.0]	6.8 [6.5 – 7.1]
**OGFR gene**	4.7 [4.1 – 5.3]	4.7 [4.2 – 5.3]	4.7 [4.2 – 5.3]	4.5 [3.9 – 5.2]
**Disease free time** (days)			975 [538 – 1677]	724 [410 – 1340]
**Relapse** (yes) % (N)			16.5 (56/340)	16.8 (531/3167)
**Overall survival time** (days)			925 [491 – 1813]	738 [402 – 1409]
**Death** (yes) % (N)			37.4 (235/629)	25.9 (1412/5443)
**AJCC stage** % (N)				
I			198 (34.9)	1660 (34.3)
II			178 (31.3)	1452 (30.0)
III			123 (21.7)	1118 (23.1)
IV			69 (12.1)	616 (12.7)
**Age at diagnosis**	62 [53 – 71]		63 [52 – 72]	62 [53 – 71]
**Gender** (Male) % (N)	53.7 (4484/8780)	59.8 (1348/2256)	48.6 (306/629)	51.8 (2830/5467)
**Primary site** % (N)				
Bladder	4.4 (390/8780)	0.5 (11/2256)	2.5 (17/672)	6.2 (362/5852)
Breast	13.5 (1181/8780)	3.9 (89/2256)	16.4 (110/672)	16.8 (982/5852)
Colon	8.7 (762/8780)	15.0 (339/2256)	7.6 (51/672)	6.4 (372/5852)
Esophagus	9.7 (853/8780)	29.2 (659/2256)	1.6 (11/672)	3.1 (183/5852)
Kidney	10.6 (929/8780)	1.4 (32/2256)	18.8 (126/672)	13.2 (771/5852)
Liver	5.2 (458/8780)	5.1 (115/2256)	7.1 (48/672)	5.0 (295/5852)
Lung	16.1 (1415/8780)	13.9 (313/2256)	16.4 (110/672)	17.0 (992/5852)
Prostate	6.6 (580/8780)	4.7 (106/2256)	7.1 (48/672)	7.3 (426/5852)
Salivary Gland	5.7 (502/8780)	0 (0/2256)	6.2 (42/672)	7.9 (460/5852)
Stomach	6.9 (605/8780)	8.5 (192/2256)	4.9 (33/672)	6.5 (380/5852)
Thyroid	9.2% (812)	14.1 (318/2256)	7.9 (53/672)	7.5 (441/5852)
Uterus	3.3% (293)	3.6 (82/2256)	3.4 (23/672)	3.2 (188/5852)

Data are reported as median [25^th^ – 75^th^ percentile] or % (N). MOR, µ opioid receptor; KOR, κ opioid receptor; DOR, δ opioid receptor; TLR4, toll–like receptor 4; OGFR, opioid growth factor receptor; Age for GTEx samples is not reported because is recorded as a categorical variable with 10 years strata and not as a continuous variable.

**Figure 3:**
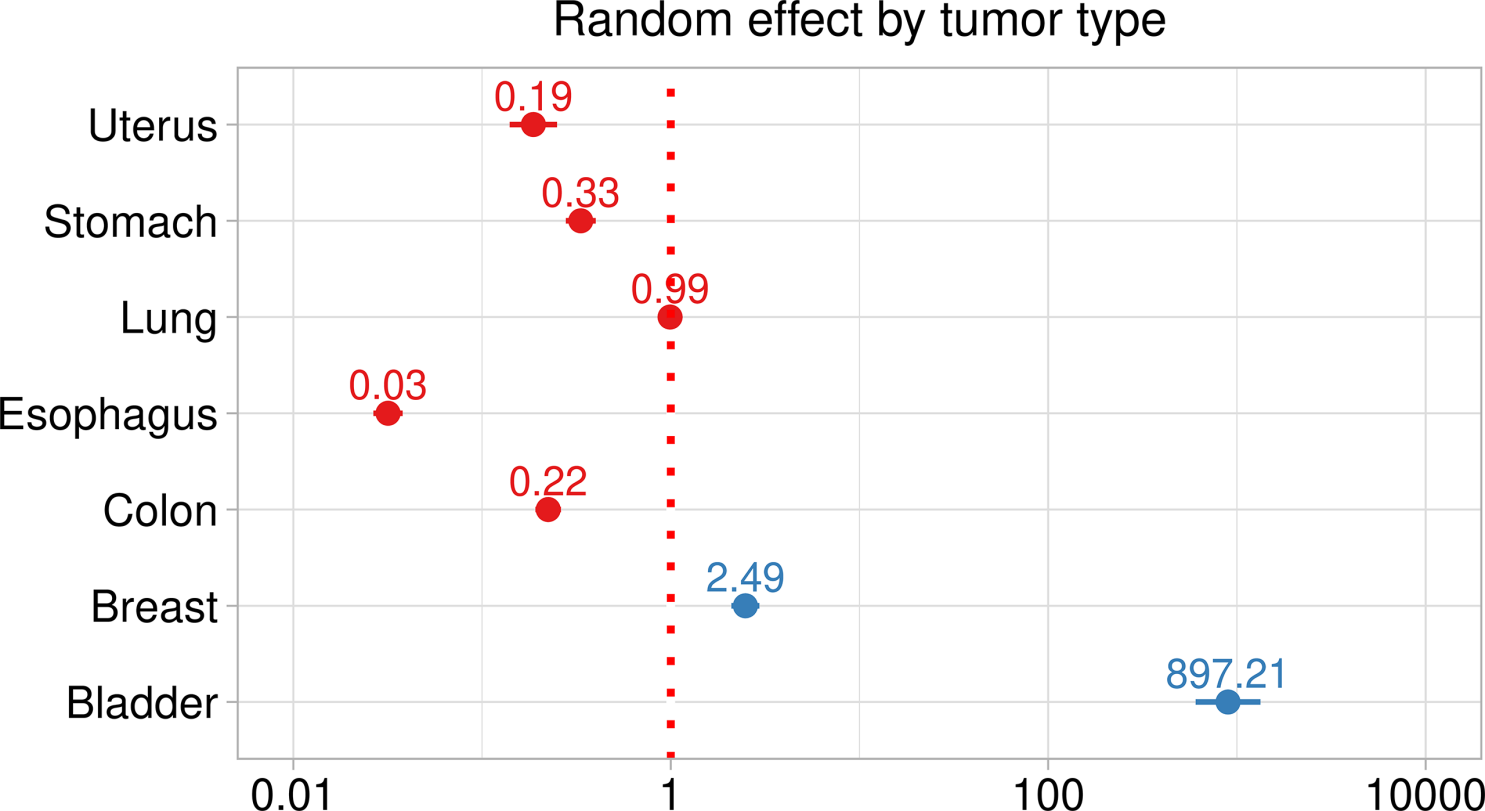
Random effect plot of the mixed logistic model assessing the association between opioid receptors expression and type of tissue. Red dotted line, significance threshold. Dots effect estimates and bar 95% Confidence intervals.

**Figure 4:**
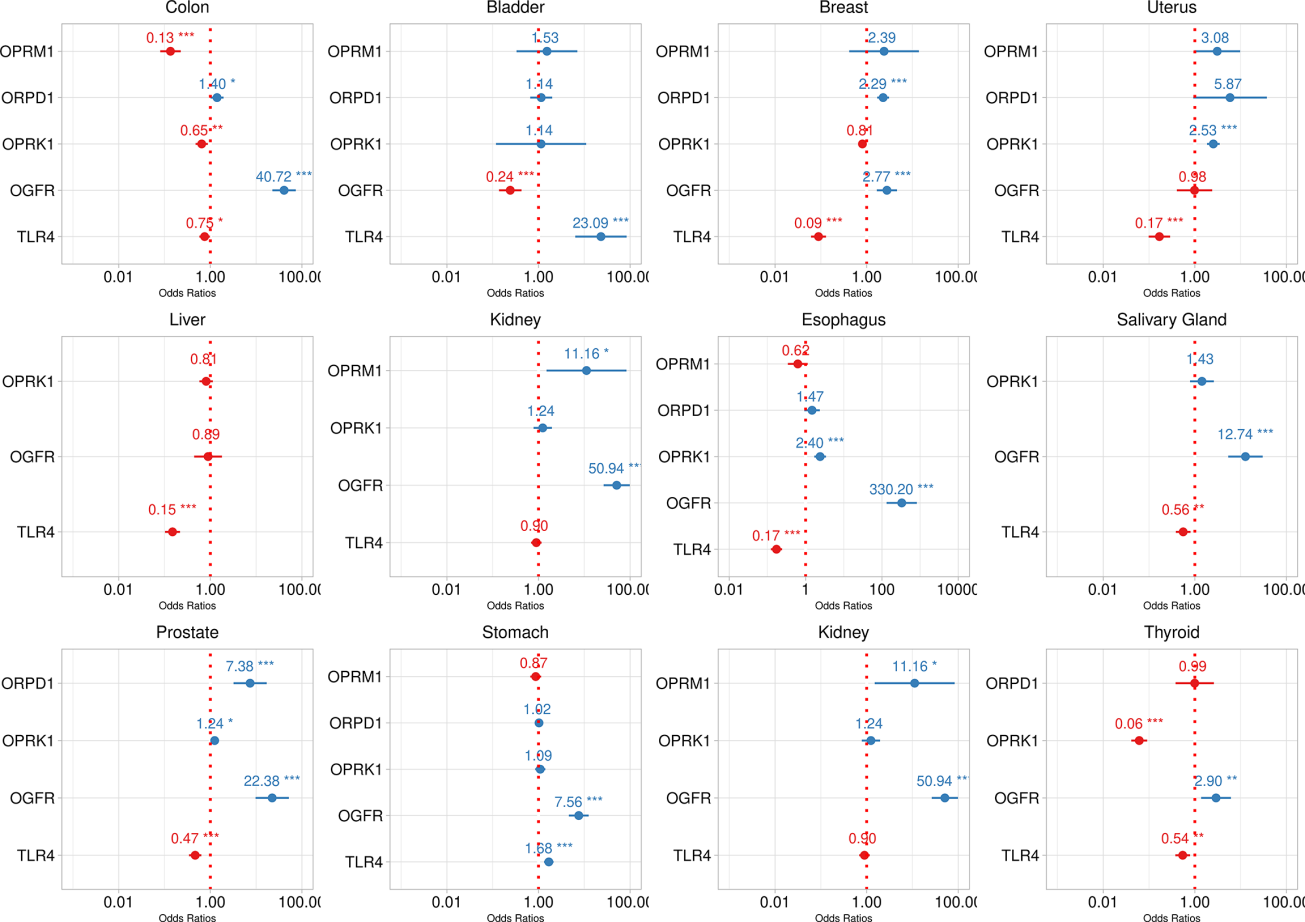
Logistic model fit of opioid receprotrs association with tumor tissue by tumor type. Dotted red line represents no effect. Estimates are reported as red or blue when the odds ratio point estimate is lower or greater than one respectively. Dots are point estimates and bars 95% confidence intervals. Statistical significance is reported as * < 0.05, ** < 0.001, *** < 0.001.

The logistic model’s estimates are reported in **Table 2**. Opioid receptors were significantly associated with tumor samples, albeit differently, as some genes associated positively and others negatively. The estimated standard deviation among tumors, i.e. 3.06, is bigger than the largest estimate among the fixed effects, i.e. OGFR estimate 2.39, suggesting considerable effect differences among tumors (**Figure 3**). Logistic models estimated for each tumor are reported in **Figure 4** and show considerable variability among opioid receptor estimates across tumor types.

**Table 2:** The association between opioid receptor gene expression and tumor type.

Gene expression (Log scale)	Odds Ratio [Lower–Upper 95%CI]	P– value
MOR	0.74 [0.63 – 0.87]	< 0.001
KOR	1.27 [1.17 – 1.37]	< 0.001
DOR	1.66 [1.48 – 1.87]	< 0.001
TLR4	0.29 [0.26 – 0.32]	< 0.001
OGFR	2.39 [2.05 – 2.78]	< 0.001
**Random effect parameter** (Tumor type): Standard deviation: 3.06
**ICC** (Tumor_type): 0.74

Primary site random effect Standard deviation for multivariable model: 1.34. MOR, µ opioid receptor; KOR, κ opioid receptor; DOR, δ opioid receptor; TLR4, toll–like receptor; OGFR, opioid growth factor receptor; CI, Confidence Interval; ICC, Intraclass correlation coefficient. The model has been estimated with all tumor types with available data for all receptors.

Mixed Cox models estimates for DFI and OS are reported in **Table 3**. After controlling for age and cancer stage, we found no association overall between opioid receptor expression and long-term outcomes except a weak effect for KOR on OS. The cancer stage is by far the predominant effect in both DFI and OS models as was expected.

**Table 3:** Opioid gene expression association with long term oncologic outcomes.

Disease Free interval		
Gene expression (Log scale)	Hazard Ratio [Lower–Upper 95%CI]	P value
**MOR**	1.17 [0.89– 1.68]	0.203
**KOR**	1.07 [0.97 –1.18]	0.156
**DOR**	1.06 [0.91 – 1.24]	0.417
**TLR4**	1.01 [0.88 – 1.14]	0.929
**OGFR**	1.02 [0.85 – 1.23]	0.765
**Age at diagnosis**	0.99 [0.98 – 1.00]	0.203
AJCC Stage (Ref Stage I)*		
II	1.32 [0.99 – 1.77]	0.057
III	1.96 [1.43 – 2.69]	> 0.001
**Random effect parameter (Tumor type): Standard deviation: 0.64**		
**Overall Survival**		
**Gene expression (Log scale)**	**Hazard Ratio [Lower–Upper 95%CI]**	**P value**
**MOR**	1.17 [0.97 – 1.41]	0.087
**KOR**	1.10 [1.01 – 1.21]	0.021
**DOR**	1.00 [0.94 – 1.07]	0.794
**TLR4**	0.98 [0.90 – 1.07]	0.731
**OGFR**	1.00 [0.90 – 1.11]	0.937
**Age at diagnosis**	1.02 [1.01 – 1.03]	< 0.001
**AJCC Stage (Ref: Stage I)**		
II	1.49 [1.22 – 1.82]	< 0.001
III	2.57 [2.10 – 3.14]	< 0.001
IV	4.69 [3.66 – 6.01]	< 0.001
**Random effect parameter (Tumor type): Standard deviation: 0.62**

MOR, µ opioid receptor; KOR, κ opioid receptor; DOR, δ opioid receptor; TLR4, toll–like receptor; OGFR, opioid growth factor receptor; CI, Confidence Interval; AJCC, American Joint Committee on Cancer. *AJCC stage IV data were not available for Disease free interval.

## Discussion

This study’s’ main findings can be summarized as follows: Firstly, higher or lower opioid receptor gene expression within tumors is variable depending on the specific tumor type; Secondly, single gene expression also varies depending on tumor type; Thirdly, there was no association between opioid gene receptor expression and DFI or OS after controlling for age and tumor stage.

This analysis has several strengths. First, to our knowledge, this is the first analysis of large public genetic databases focusing specifically on tumor opioid receptor expression and their link to cancer outcome. Secondly, we selected normalized data through a previously published meticulous procedure that consistently removes the batch effect from samples. Thirdly, we controlled for confounding bias by performing a time–to–event analysis, including potential confounders such as age and tumor stage. We also assessed the effect of specific cancer types by adding a random effect to the multivariable model to account for the hierarchical structure of the data. Fourthly, we included all previously studied opioid receptors known to be involved in perioperative opioid drug binding in our analysis.

The role of tumor opioid receptor expression on tumor growth and metastasis has generated considerable interest among researchers involved in surgical oncology (1). Because of the pivotal role of opioid analgesics in the perioperative process, it has been speculated that activation of these receptors could cause cancer cells to proliferate, migrate and escape immune control. Our findings add further to previous studies assessing opioid receptor expression in different tumors. Higher MOR expression was found on prostate cancer samples compared to unpaired control tissue (22). Likewise, in a study that compared human lung cancer samples with non-tumor adjacent tissue samples, MOR expression was significantly increased in tumor tissue from patients with metastatic lung cancer had an approximately twofold increase in MOR expression (21).

Higher expression of MOR was associated with tumor tissue in gastric cancer, hepatocellular carcinoma, and colorectal cancer samples (17–20, 24). In contrast, this association of MOR-1 with oncological results was not observed in other tumors. For instance, in esophageal squamous cell carcinoma (ESCC), MOR expression in the cytoplasm was associated with lymph node metastases. However, no link was found between MOR expression and the OS of patients with ESCC (18).

In a retrospective analysis among breast cancer patients, which analyzed the effect of anesthetic technique on MOR expression, the authors found that general anesthesia with opioid analgesia increased MOR expression in the resected tumor compared to anesthetic technique with locoregional analgesia (30), These results support the hypothesis that the opioid receptor genetic footprint varies with tumor type. This is consistent with recent data from a triple-negative breast tumor databank, which analyzed the same receptors as us and found that MOR, OPRD, and OPRK were overexpressed, while TLR4 was downregulated. Furthermore, these authors found that higher doses of intraoperative opioids were associated with somewhat worse oncologic outcomes than patients receiving lower doses during surgery (16). A thorough mapping of different receptors’ expression is important because opposing effects have been described, with some receptor activation having protumor effects while others have potentially tumor suppressing effects. This is even more important since both exogenous and endogenous opioid receptor agonists may play a different role depending on the specific profile of receptor expression, while opioid receptor antagonists such as methylnaltrexone have found to be associated with longer median survival in an unplanned posthoc analysis of two clinical trials (31).

We found no association between opioid receptor expression and long-term outcomes such as DFI and OS. Existing data on this matter are diverging. For example, while some studies found an association between MOR expression in particular and cancer recurrence (18, 19, 22), others did not (16, 24). Indeed, available research on this topic seems to point out that there is no one-size-fits-all explanation to this question. Further investigations on the specific receptor profile of each cancer strain should lay the foundation on whether opioid receptors can be included as oncologic prognostic markers.

Studies that assess opioid receptor expression in cancer rely on immunohistochemistry assay, while quantitative methods such as quantitative real-time polymerase chain reaction (qRT–PCR) are seldom reported, and with different procedures and primers (**eTable 1 Supplementary Material**) (15, 19, 20). Since there are no consensus guidelines on how to perform these assays (32), we consider that availing of large and validated databases such as the TCGA and GTEx is a powerful tool to draw a genetic footprint of opioid receptors in tumor cells. Furthermore, as our present results suggest, the impact of opioid receptors on cancer cells does not seem to be based on a simple pathway involving an individual receptor overexpression and is probably a more integrated mechanism involving several receptor targets with different effects that can vary depending on specific tumor type.

Furthermore, genetic content within tumors is variable and opioid receptors can present single nucleotide polymorphisms (SNPs). This type of polymorphisms on known oncogenes such as p53 and X-Ray Repair Cross Complementing 3 (XRRCC3) genes have been studied to elucidate their effect on cancer susceptibility with conflicting results to date (33–35). For instance, particular SNPs such as the A118G have been linked to reduced sensitivity to opioid medication in the patient suffering from chronic pain (36, 37) and cancer (19) and even cancer recurrence in specific tumor types and populations (38, 39), Also, TLR4 gene polymorphisms have also been studied and may play a role in proliferation and differentiation and multiple isoforms of receptor subtype resulting from alternative splicing of the pre-mRNA transcript have been identified albeit their functional role has yet to be clarified (40). Investigators are beginning to expand the horizon outside the genetic profile of opioid receptors and to include specific genetic alterations such as Cyclin-Dependent Kinase Inhibitor 2A (CDKN2A) mutations (41), although the influence that specific influence of individual receptor isoforms is still a matter of debate (42).

Several limitations must be acknowledged. For instance, differences in baseline characteristics from GTEx and TCGA repositories may be present. Also, although we controlled for age and cancer stage, the effect of other confounding factors not included in the analysis, such as type of surgery or pathologic stage or opioid agonists or antagonists administration, cannot be ruled out. These parameters could have a modifying effect on the association between opioid receptor expression and long-term cancer outcomes. Furthermore, we also acknowledge that while we assessed all the most common opioid receptor genes, other molecular pathways can be involved in the effect of opioids on cancer growth. Finally, because of the hypothesis-generating purpose of this study, we did not set any *a priori* effect threshold or multiple comparisons correction; hence some results’ statistical significance and the potential hypothesis derived from them should be confirmed in future trials.

In conclusion, the most common solid tumors express higher opioid receptor genes than normal tissue, but variably depending on the primary tumor analyzed. No association was found between disease-free and overall survival and opioid gene expression after controlling for age and tumor stage. Further studies are warranted to elucidate the specific genetic footprint of opioid receptors in each cancer type and the potential role of gene polymorphisms.

## Data Availability Statement

Publicly available datasets were analyzed in this study. This data can be found here: https://www.nature.com/articles/sdata201861#ref-CR22.

## Authors Contributions

AB: This author conceived the idea, helped with data acquisition, critical review of the content, and manuscript preparation. SZ: This author coded the data acquisition from the repositories, provided a critical review of the content, and helped with manuscript preparation. IG-C: This author provided a critical review of the content, and helped with manuscript preparation. PE: This author provided a critical review of the content, and helped with manuscript preparation. MA: This author provided a critical review of the content, and helped with manuscript preparation. DB: This author provided a critical review of the content, and helped with manuscript preparation. OD-C: This author provided a critical review of the content, and helped with manuscript preparation. GM: This author conceived the idea, helped with data acquisition, critical review of the content, and manuscript preparation. All authors contributed to the article and approved the submitted version.

## Conflict of Interest

OD-C: Received payment for educational talks and scientific conferences from MSD (Merck Sharp & Dohme, Inc.).

The remaining authors declare that the research was conducted in the absence of any commercial or financial relationships that could be construed as a potential conflict of interest.

## Publisher’s Note

All claims expressed in this article are solely those of the authors and do not necessarily represent those of their affiliated organizations, or those of the publisher, the editors and the reviewers. Any product that may be evaluated in this article, or claim that may be made by its manufacturer, is not guaranteed or endorsed by the publisher.

## References

[1] Sullivan R Alatise OI Anderson BO Audisio R Autier P Aggarwal A. Global Cancer Surgery: Delivering Safe, Affordable, and Timely Cancer Surgery. Lancet Oncol (2015) 16:1193–224. doi: 10.1016/S1470-2045(15)00223-5 26427363

[2] Peirs C Seal RP. Neural Circuits of Pain: Recent Advances and Current Perspectives. Science (2016) 354:578–84. doi: 10.1126/science.aaf8933 PMC11327866 27811268

[3] Stein C. The Control of Pain in Peripheral Tissue by Opioids. N Engl J Med (1995) 332(25):1685–90. doi: 10.1056/NEJM199506223322506 7760870

[4] Waldhoer M Bartlett SE Whistler JL. Opioid Receptors. Annu Rev Biochem (2004) 73:953–90. doi: 10.1146/annurev.biochem.73.011303.073940 15189164

[5] Hollmann MW Rathmell JP Lirk P. Optimal Postoperative Pain Management: Redefining the Role for Opioids. Lancet (2019) 393(10180):1483–5. doi: 10.1016/S0140-6736(19)30854-2 30983573

[6] Brat GA Agniel D Beam A Yorkgitis B Bicket M Homer M. Postsurgical Prescriptions for Opioid Naive Patients and Association With Overdose and Misuse: Retrospective Cohort Study. BMJ (2018) 360:j5790. doi: 10.1136/bmj.j5790 29343479 PMC5769574

[7] Neuman MD Bateman BT Wunsch H. Inappropriate Opioid Prescription After Surgery. Lancet (2019) 393:1547–57. doi: 10.1016/S0140-6736(19)30428-3 PMC6556783 30983590

[8] Makary MA Overton HN Wang P. Overprescribing is Major Contributor to Opioid Crisis. BMJ (2017) 359:j4792. doi: 10.1136/bmj.j4792 29051174

[9] Boland JW McWilliams K Ahmedzai SH Pockley AG. Effects of Opioids on Immunologic Parameters That are Relevant to Anti-Tumour Immune Potential in Patients With Cancer: A Systematic Literature Review. Br J Cancer (2014) 111:866–73. doi: 10.1038/bjc.2014.384 PMC4150281 25025960

[10] Wigmore T Farquhar-Smith P. Opioids and Cancer: Friend or Foe? Curr Opin Support Palliat Care (2016) 10:109–18. doi: 10.1097/SPC.0000000000000208 26990052

[11] Sekandarzad MW Van Zundert AAJ Lirk PB Doornebal CW Hollmann MW. Perioperative Anesthesia Care and Tumor Progression. Anesth Analg (2017) 124:1697–708. doi: 10.1213/ANE.0000000000001652 27828796

[12] Kim JY Ahn HJ Kim JK Kim J Lee SH Chae HB. Morphine Suppresses Lung Cancer Cell Proliferation Through the Interaction With Opioid Growth Factor Receptor: An *In Vitro* and Human Lung Tissue Study. Anesth Analg (2016) 123:1429–36. doi: 10.1213/ANE.0000000000001293 27167686

[13] Liao SJ Zhou YH Yuan Y Li D Wu FH Wang Q. Triggering of Toll-Like Receptor 4 on Metastatic Breast Cancer Cells Promotes αvβ3-Mediated Adhesion and Invasive Migration. Breast Cancer Res Treat (2012) 133:853–63. doi: 10.1007/s10549-011-1844-0 22042369

[14] Matzner P Sorski L Shaashua L Elbaz E Lavon H Melamed R. Perioperative Treatment With the New Synthetic TLR-4 Agonist GLA-SE Reduces Cancer Metastasis Without Adverse Effects. Int J Cancer (2016) 138:1754–64. doi: 10.1002/ijc.29885 PMC4724303 26453448

[15] Jorand R Biswas S Wakefield DL Tobin SJ Golfetto O Hilton K. Molecular Signatures of Mu Opioid Receptor and Somatostatin Receptor 2 in Pancreatic Cancer. Mol Biol Cell (2016) 27:3659–72. doi: 10.1091/mbc.E16-06-0427 PMC5221597 27682590

[16] Montagna G Gupta HV Hannum M Tan KS Lee J Scarpa JR. Intraoperative Opioids are Associated With Improved Recurrence-Free Survival in Triple-Negative Breast Cancer. Br J Anaesth (2021) 126:367–76. doi: 10.1016/j.bja.2020.10.021 PMC8014943 33220939

[17] Nylund G Pettersson A Bengtsson C Khorram-Manesh A Nordgren S Delbro DS. Functional Expression of μ-Opioid Receptors in the Human Colon Cancer Cell Line, HT-29, and Their Localization in Human Colon. Dig Dis Sci (2008) 53:461–6. doi: 10.1007/s10620-007-9897-y 17680363

[18] Zhang YF Xu QX Liao LD Xu XE Wu JY Wu ZY. Association of Mu-Opioid Receptor Expression With Lymph Node Metastasis in Esophageal Squamous Cell Carcinoma. Dis Esophagus (2015) 28:196–203. doi: 10.1111/dote.12165 24428760

[19] Yao YS Yao RY Zhuang LK Qi WW Lv J Zhou F. MOR1 Expression in Gastric Cancer: A Biomarker Associated With Poor Outcome. Clin Transl Sci (2015) 8:137–42. doi: 10.1111/cts.12246 PMC5350983 25441763

[20] Chen DT Pan JH Chen YH Xing W Yan Y Yuan YF. The Mu-Opioid Receptor is a Molecular Marker for Poor Prognosis in Hepatocellular Carcinoma and Represents a Potential Therapeutic Target. Br J Anaesth (2019) 122:e157–67. doi: 10.1016/j.bja.2018.09.030 30915986

[21] Singleton PA Mirzapoiazova T Hasina R Salgia R Moss J. Increased μ-Opioid Receptor Expression in Metastatic Lung Cancer. Br J Anaesth (2014) 113:i103–8. doi: 10.1093/bja/aeu165 PMC4111280 24920011

[22] Zylla D Gourley BL Vang D Jackson S Boatman S Lindgren B. Opioid Requirement, Opioid Receptor Expression, and Clinical Outcomes in Patients With Advanced Prostate Cancer. Cancer (2013) 119:4103–10. doi: 10.1002/cncr.28345 PMC3833881 24104703

[23] Zhang H Sun M Zhou D Gorur A Sun Z Zeng W. Increased Mu-Opioid Receptor Expression is Associated With Reduced Disease-Free and Overall Survival in Laryngeal Squamous Cell Carcinoma. Br J Anaesth (2020) 125:722–9. doi: 10.1016/j.bja.2020.07.051 32900505

[24] Díaz-Cambronero O Mazzinari G Giner F Belltall A Ruiz-Boluda L Marqués-Marí A. Mu Opioid Receptor 1 (MOR-1) Expression in Colorectal Cancer and Oncological Long-Term Outcomes: A Five-Year Retrospective Longitudinal Cohort Study. Cancers (Basel) (2020) 12(1):134. doi: 10.3390/cancers12010134 PMC7016725 31948099

[25] Grossman RL Heath AP Ferretti V Varmus HE Lowy DR Kibbe WA. Toward a Shared Vision for Cancer Genomic Data. N Engl J Med (2016) 375:1109–12. doi: 10.1056/nejmp1607591 PMC6309165 27653561

[26] Tomczak K Czerwińska P Wiznerowicz M. The Cancer Genome Atlas (TCGA): An Immeasurable Source of Knowledge. Contemp Oncol (Pozn) (2015) 19:A68–77. doi: 10.5114/wo.2014.47136 PMC4322527 25691825

[27] Wang Q Armenia J Zhang C Penson AV Reznik E Zhang L. Data Descriptor: Unifying Cancer and Normal RNA Sequencing Data From Different Sources. Sci Data (2018) 5:180061. doi: 10.1038/sdata.2018.61 29664468 PMC5903355

[28] Colaprico A Silva TC Olsen C Garofano L Cava C Garolini D. TCGAbiolinks: An R/Bioconductor Package for Integrative Analysis of TCGA Data. Nucleic Acids Res (2016) 44:e71. doi: 10.1093/nar/gkv1507 26704973 PMC4856967

[29] Buggy DJ Freeman J Johnson MZ Leslie K Riedel B Sessler DI. Systematic Review and Consensus Definitions for Standardised Endpoints in Perioperative Medicine: Postoperative Cancer Outcomes. Br J Anaesth (2018) 121:38–44. doi: 10.1016/j.bja.2018.03.020 29935592

[30] Levins KJ Prendeville S Conlon S Buggy DJ. The Effect of Anesthetic Technique on µ-Opioid Receptor Expression and Immune Cell Infiltration in Breast Cancer. J Anesth (2018) 32:792–6. doi: 10.1007/s00540-018-2554-031 PMC6267716 30229370

[31] Janku F Johnson LK Karp DD Atkins JT Singleton PA Moss J. Treatment With Methylnaltrexone is Associated With Increased Survival in Patients With Advanced Cancer. Ann Oncol (2016) 27:2032–8. doi: 10.1093/annonc/mdw317 PMC6267944 27573565

[32] Du KN Feng L Newhouse A Mehta J Lasala J Mena GE. Effects of Intraoperative Opioid Use on Recurrence-Free and Overall Survival in Patients With Esophageal Adenocarcinoma and Squamous Cell Carcinoma. Anesth Analg (2018) 127:210–6. doi: 10.1213/ANE.0000000000003428 29757780

[33] Kuang J Yan X Genders AJ Granata C Bishop DJ. An Overview of Technical Considerations When Using Quantitative Real-Time PCR Analysis of Gene Expression in Human Exercise Research. PloS One (2018) 13(5):1–27. doi: 10.1371/journal.pone.0196438 PMC5944930 29746477

[34] Manuguerra M Saletta F Karagas MR Berwick M Veglia F Vineis P. XRCC3 and XPD/ERCC2 Single Nucleotide Polymorphisms and the Risk of Cancer: A HuGE Review. Am J Epidemiol (2006) 164:297–302. doi: 10.1093/aje/kwj189 16707649

[35] Chen X Liu F Li B Wei YG Yan LN Wen TF. P53 Codon 72 Polymorphism and Liver Cancer Susceptibility: A Meta-Analysis of Epidemiologic Studies. World J Gastroenterol (2011) 17:1211–8. doi: 10.3748/wjg.v17.i9.1211 PMC3063916 21448428

[36] Kasai S Ikda K. Pharmacogenomics of the Human µ-Opioid Receptor Pharmacogenomics. Pharmacogenomics (2011) 12:1305–20. doi: 10.2217/pgs.11.68 21919606

[37] Janicki PK Schuler G Francis D Bohr A Gordin V Jarzembowski T. A Genetic Association Study of the Functional A118G Polymorphism of the Human μ-Opioid Receptor Gene in Patients With Acute and Chronic Pain. Anesth Analg (2006) 103:1011–7. doi: 10.1213/01.ane.0000231634.20341.88 17000822

[38] Bortsov AV Millikan RC Belfer I Boortz-Marx RL Arora H Mclean SA. μ-Opioid Receptor Gene A118G Polymorphism Predicts Survival in Patients With Breast Cancer. Anesthesiology (2012) 116:896–902. doi: 10.1097/ALN.0b013e31824b96a1 22433205 PMC3310356

[39] Wang S Li Y Liu XD Zhao CX Yang KQ. Polymorphism of A118G in μ-Opioid Receptor Gene is Associated With Risk of Esophageal Squamous Cell Carcinoma in a Chinese Population. Int J Clin Oncol (2013) 18:666–9. doi: 10.1007/s10147-012-0441-5 22752309

[40] Regan PM Langford D Khalili K. Regulation and Functional Implications of Opioid Receptor Splicing in Opioid Pharmacology and HIV Pathogenesis. J Cell Physiol (2016) 231(5):976–85. doi: 10.1002/jcp.25237 PMC4728022 26529364

[41] Connolly JG Tan KS Mastrogiacomo B Dycoco J Caso R Jones GD. Intraoperative Opioid Exposure, Tumour Genomic Alterations, and Survival Differences in People With Lung Adenocarcinoma. Br J Anaesth (2021) 127:75–84. doi: 10.1016/j.bja.2021.03.030 34147159 PMC8258974

[42] Scroope CA Singleton Z Hollmann MW Parat MO. Opioid Receptor-Mediated and Non-Opioid Receptor-Mediated Roles of Opioids in Tumour Growth and Metastasis. Front Oncol (2021) 11:792290:792290. doi: 10.3389/fonc.2021.792290 35004315 PMC8732362

