# Supplementary material for: Solid Tumor Opioid Receptor Expression and Oncologic Outcomes: Analysis of the Cancer Genome Atlas and Genotype Tissue Expression Project
Source: Front Oncol. 2022 Mar 10;12:801411. doi: 10.3389/fonc.2022.801411 (PMC8960174; doi:10.3389/fonc.2022.801411)
Supplement: Supplementary file 1 [file Table_1.docx]

**Solid Tumor Opioid Receptor Expression and Oncologic Outcomes:**

**Analysis of the Cancer Genome Atlas and Genotype Tissue Expression Project**

**SUPPLEMENTARY DIGITAL CONTENT**

*Belltall A^1,2^, Zúñiga-Trejos S^3^, Garrido-Cano I^4,6^, Eroles P^4,6^, Argente-Navarro MP ^1,2^, Buggy DJ^5,6^, Díaz-Cambronero O^1,2,6^, Mazzinari G^1,2,6^

^1^ Perioperative Medicine Research Group. Instituto de Investigación Sanitaria la Fe. Valencia, Spain;

^2^Departament de Anesthesiology. Hospital Universitari i Politécnic la Fe. Valencia, Spain;

^3^ Bioinformatics and Biostatistics Unit, INCLIVA Biomedical Research Institute, Valencia, Spain;

^4^ Breast cancer research group, Molecular and cellular Oncolgy unit, Biomedical Research Institute. INCLIVA. Valencia,Spain;

^5^Department of Anaesthesiology & Perioperative Medicine, Mater University Hospital, University College Dublin, Dublin, Ireland;

^6^Euro-Periscope: The Onco-Anaesthesiology Research Group (RG) of European Society of Anaesthesiology & Intensive Care (ESA-IC), Brussels, Belgium

| **eTable 1.** Overview of previous studies on MOR expression in different type of cancers | | | | | |
| --- | --- | --- | --- | --- | --- |
| **Study** | **Study Population** | **Cell culture** | **Control type** | **IHC technique** | **PCR Technique** |
| **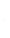Montagna (2020)**  **British Journal of Anaesthesia**  **DOI: 10.1016/j.bja.2020.10.021** | Stage I to III triple negative breast cancer | Samples from Cancer Genome Atlas | Normal breast tissue (supposedly form the TCGA) |  | To assess cellular-level expression of the opioid receptor in specific cell types in the TME, a single-cell RNA-seq data set produced by Karaayvaz and colleagues was used, and 1112 clustered cells from primary TNBC tumours were analysed. Expression data for the specific genes of interest were extracted from the processed data therein (wich were therefore already normalised and log2-transformed as described in step 2 of their methodology, see Supplementary material of Karaayvaz colleagues). |
| **Diaz–Cambronero (2020)**  **Cancers**  **DOI: 10.3390/cancers12010134** | Stage II to III  Colorectal cancer | None | Corresponding non–tumor tissue samples | Sections were heated in an Envision Flex bu er (pH = 9) for 20 min and incubated for 30 min at room temperature with a mouse monoclonal MOR-1 antibody (1:100) (Acris®). |  |
| **Nylund (2008)**  **Digestive Diseases and Science**  **DOI: 10.1007/s10620-007-9897-y** | Dukes B-C  Colon Cancer | HT–29 cell line | Macroscopically tumor free bowel wall | Primary antibody (a goat polyclonal anti-MOR-1 antibody to an epitope mapping within the N-terminal of the extracellular domain of MOR-1 of human origin [1:100 or 1:200], or a rabbit polyclonal anti-MOR-1 antibody to an epitope corresponding to amino acids 1-80 mapping at the N-terminus of MOR-1 of human origin [1:50, 1:100, or 1:200], both from Santa Cruz Biotechnology; Santa Cruz, Calif). |  |
| **Zhang (2015)**  **Disease of esophagus**  **DOI: 10.1111/dote.12165** | Esophagus squamous cell carcinoma | KYSE180, KYSE150, and EC109 cell lines | None | Polyclonal rabbit anti-MOR 1 (1:50, Abgent, San Diego, CA, USA).  Poly-HRP Anti-Mouse/Rabbit IgG Detection System (ZSGB-BIO, Beijing, China) and the Liquid DAB Substrate Kit (Invitrogen, Carlsbad, CA, USA).  Normal rabbit IgG (1:1000, Santa Cruz Biotechnology) and phosphate buffer solution were used as negative controls for primary antibody. |  |
| **Yao (2014)**  **Clinical and translational science**  **DOI: 10.1111/cts.12246** | Gastric Cancer | None | 93 paracancer tissues which were more than 5 cm away from the edge of tumor were randomly selected. | Rabbit polyclonal antibody against human MOR1 (dilution 1:100, Santa Cruz Biotechnology, Dallas, TX, USA). | Total RNA was extracted using TRIzol reagent (Invitrogen, Tokyo, Japan), and 5 mg of extracted RNA samples was reverse transcribed into cDNA by a One-step PrimescrioptcDNA Synthesis Kit (TaKaRa, Dalian, China) according to the manufacture’s protocol (Promega, Madison, Wi, USA). GAPDH was used for normalization. Primers for MOR1 and GAPDH were from GeneCopoeia (HQP012059, HQP064347). |
| **Chen (2018)**  **British Journal of Anaesthesia**  **DOI: 10.1016/j.bja.2018.09.030** | Hepatocellular carcinoma | MHCC-97L; MHCC-97H; QGY-7703;  Huh7;  HepG2;  LO2 | Corresponding non-tumour tissue samples | Anti-MOP antibody (NBP1-930, NOVUS), anti-E-cadherin antibody (ZM-0092, ZSGB-BIO), and anti-Vimentin antibody (5741, CST) at a 1:200 dilution. Afterwards, the EnVision kit (Dako Cytomation, Carpinteria, USA) was used to detect the antibodies in the tissue sections. | For the qRT-PCR analysis, we used SYBR Green qPCR SuperMix (Invitrogen, USA) and the CFX96™ Real-Time PCR Detection System (Bio-Rad, USA). The housekeeping gene GAPDH was used as the endogenous control. Values herein are the mean of triplicates in three independent experiments. |
| **Zylla (2013)**  **10.1002/cncr.28345**  **2013**  **Cancer** | Stage IV prostate cancer | None | Samples of benign prostatic  Hyperplasia (BPH) used as controls were obtained from patients without prostate cancer | 1:100 guinea pig anti-human MOR (Millipore, Billerica, MA) and 2º donkey anti-guinea pig Cy5 (1:200, Jackson Immunoresearch, West Grove, PA).  In parallel, sections were stained with isotype-matched immunoglobulin G and 2º antibody for negative controls. |  |
| **Zhang (2020)**  **British Journal of Anesthesia**  **10.1016/j.bja.2020.07.051**  **2020** | Laryngeal squamous cell carcinoma | Samples from Cancer Genome Atlas | None just Higher MOR and lower MOR arbitrarily | Anti-MOR antibody (UMB3)-C-terminal (ab134054). The secondary antibody was goat anti-rabbit IgG H&L (HRP) (ab205718). |  |
| **Jorand (2016)**  **Molecular Biology of the cell**  **DOI: 10.1091/mbc.E16-06-0427**  **2016** | Pancreatic cancer | PANC-1; BxPC-3; SU.86.86; CAPAN-1 | Healthy pancreas | Anti-MOR (guinea pig polyclonal; Abcam, Cambridge, MA). Purified secondary antibody rabbit anti-guinea pig (polyclonal; Abcam). | MOR-forward (5’-TCTGGCTCCAAAGAAAAGGA-3’) and MOR-reverse (5’-CAATGCAGAAGTGCCAAGAA-3’) primers. |
| **Singleton (2014)**  **British Journal of Anesthesia**  **DOI:10.1093/bja/aeu165** | Non-small cell lung cancer | None | Corresponding non–tumor tissue samples | Rabbit anti-MOR antibody (GeneTex, San Antonio, TX, USA) |  |
